# Leader’s strategies for designing the promotional path of regional brand competitiveness in the context of economic globalization

**DOI:** 10.3389/fpsyg.2022.972371

**Published:** 2022-08-11

**Authors:** Pei Li, Jianguo Du, Fakhar Shahzad

**Affiliations:** School of Management, Jiangsu University, Zhenjiang, China

**Keywords:** sustainable strategy, brand competitiveness, brand market, government guidance, resource capacity

## Abstract

In the era of economic globalization, the competitiveness of products on a global scale is increasingly achieved through effective and sustainable strategies for brand development by the leaders. This paper conducts an empirical study on regional brand competitiveness (BC) influencing factors. A research model was proposed and tested by employing structural equation modeling. Data analysis was conducted using 214 valid questionnaires from two major producing areas in Jilin Province, China. Research results show that Brand Market (BM) and Government Guidance (GG) directly and positively impact the regional BC. Regional Resource (RR) and industrial development (ID) indirectly impact the regional BC through the mediating role of BM and GG. BM is the most important factor affecting the regional BC. Based on this, the path to improve the competitiveness of traditional agricultural products under economic globalization is determined, and targeted countermeasures and suggestions are formulated for the existing problems.

## Introduction

The general trend of world economic development entails economic globalization. Economic globalization may bring both opportunities and challenges to the agriculture of the states integrated into the world economy (Liu and Wang, 2022). In the process of economic globalization, the competitiveness of a country’s agricultural products is mainly reflected in its brand competition, for agricultural product brands carry great economic and social value (Wang et al., 2022). The top places of “World Top 500 Brands” published each year since 2004 by the World Brand Lab have always been occupied by brands (including agricultural brands) of developed countries in Europe, America, and Japan. Traditional advantageous characteristic agricultural products stand for the image and national strength of a country. Bands like the Bordeaux Wine of France, the Sunkist Orange of America, Dutch Tulips, and the Omi Beef of Japan, have all become the cultural symbols of the original countries and realize their value-added premium due to their brand effect.

Customers’ impression of brand difference increases loyalty through purchasing preferences and decisions in highly competitive marketplaces (Romaniuk et al., 2007). The studies also investigated the structures of brand marketing and strategic positioning but did not consider their combined impact on BC (Winzar et al., 2018). Pursuing “brand” has transformed the concept of consumption, making the brand the core competitiveness of firms in the face of harsh market rivalry (Liu and Wang, 2022). Green brands have come a long way in recent years, especially with the rise of e-commerce, and their influence on consumer intentions is growing (Xu et al., 2021). Agricultural food brands have grown in popularity as modern agriculture and green agriculture. Opinion leaders frequently use different promotion techniques based on brand preference. Maximizing online marketing tactics based on leader attitude disparities is a pressing issue for agricultural product brand firms to address (Liao et al., 2021). The research objects in this study are agricultural product brand firms and bounded rational leaders toward the BC. Under the pressure of the overwhelming first-mover advantage of agricultural product brands of developed countries, those developing or emerging countries are invariably confronted with a crisis of agricultural brands (Liu and Wang, 2022). Generally, there are two impediments to the traditional advantageous characteristic agricultural products of these countries: One is “products without quotation,” where countries with a large number of high-quality traditional advantageous characteristic agricultural products are reduced to exporters of raw materials for lack of brand competitiveness (BC).

The traditional advantageous and characteristic agricultural products of some developing countries have no brand recognition at all, cannot be sold on a large scale, cannot enter the industrial chain, and cannot achieve basic commercial value (Harrison et al., 2017; Wan Ismail et al., 2022). However, as economic globalization has grown, China’s traditional advantages in agricultural products have begun to feel the painful impact of first-mover advantage in agricultural product branding (Long, 2021). For example, in the international market, the two most representative traditional advantageous characteristic agricultural products in China – Changbaishan ginseng and Jilin sika deer antler – have always been facing the impact and challenges of similar industries in South Korea and New Zealand. China, South Korea, and New Zealand are all members of the Regional Comprehensive Economic Partnership (RCEP) (RECP, 2022). China’s ginseng output ranks first globally, but its output value and product price have always been at the low end of the value chain. The implementation of the RCEP will make this impact and challenge more intense.

What causes the passive situation of the Chinese ginseng and pilose antler industries? Scholars have explored the underlying reasons. Gao and Wang (2006) fixed the lack of BC of Chinese ginseng and pilose antler as an important factor. Gong (2019) noted that the low visibility of the brand of “Changbaishan Ginseng” in the international market and the unbound competition and involution of the ginseng industry in the domestic market are two conspicuous reasons. Shen et al. (2015) ascribed the reason to the long-standing problem of lacking knockout products and competitive brands in China’s deer industry. Then how do we meet the challenges posed by globalization to traditional agricultural products? This paper is aimed at exploring the influencing factors and promotion path of the BC of traditional advantageous characteristic agricultural products against the backdrop of economic globalization. First, based on previous studies, we worked out the influencing factors and establish the theoretical framework for promoting the BC of traditional advantageous characteristic agricultural products; then, the main dimensions of the influencing factors of the BC of traditional advantageous characteristic agricultural products are identified on the basis of the qualitative interviews on the two typical brands of “Changbaishan Ginseng” and “Jilin Sika Deer” in Jilin, China, with a view to constructing the theoretical framework that would explain the relationship between the variables of “regional BC of traditional advantageous characteristic agricultural products”; last but not the least, according to data collected from the questionnaire survey on the brand operation organizations and stakeholders of “Changbaishan Ginseng” and “Jilin Sika Deer” in Jilin, China, this paper conducted an empirical analysis on the conceptual model and research hypotheses on the “regional BC of traditional advantageous characteristic agricultural products.”

The main purpose of this paper is to provide empirical research answers to the following questions:

(1) What are the main factors affecting the regional BC of traditional advantageous characteristic agricultural products?
(2) How do RR endowment and ID factors affect the BC of traditional advantageous characteristic agricultural products?
(3) How can BM influence and GG and services play a mediating role between RR, industrial development, and BC?
(4) What is the path to enhancing the regional BC of traditional advantageous characteristic agricultural products?

The rest of this paper is organized as follows: Section “Theoretical background and research hypotheses” comprises a theoretical background and research hypotheses. The third section discusses the research methodology and the process objectives to be attained. Section “Results” is devoted to results and analysis, which shall be discussed in detail in Section “Discussion.” In Section “Research conclusions and countermeasures,” conclusions are drawn with countermeasures suggested.

## Theoretical background and research hypotheses

### Theoretical background

#### Research on the theoretical basis for brand competitiveness of agricultural products

Regional BC for agricultural products is a comprehensive concept. From the perspective of the basic form of market competition, regional differences are the premise for forming competitive advantages (Stoklasa and Matušínská, 2022). The theoretical premise of the regional BC research of agricultural products is the related theory of how to obtain a competitive advantage through differences (Lu et al., 2020). Therefore, the theorizing of comparative advantage and competitive advantage becomes the research basis for regional BC of agricultural products. As the main body, the market is a social network formed by consumers, enterprises, the public, and the government. The formation of agricultural BC depends on the support and efforts of these related entities (Perkins et al., 2020). Therefore, stakeholder theory is also indispensable in BC’s agricultural product area. The public attribute of the regional brand of agricultural products also involves the research of public product theory.

Ricardo (1821) put forward the theory of comparative advantage based on Adam Smith’s absolute advantage theory. It is believed that any country participating in trade will have certain advantages or disadvantages in production, but as long as the degree of advantage or disadvantage varies, the two countries can benefit from each other through the international division of labor and trade. In Brecher and Ehsan (1993) put forward the theory of factor endowment, claiming that the difference in factor endowment between two regions or countries is the main reason for the division of labor and trade between them (Chen, 2007). Helpman and Krugman (1985) analyzed the comparative advantage from the perspective of the economy of scale, proposing that comparative advantage is internal in most cases, that is, obtained through effort rather than through the endowment advocated by the traditional comparative advantage theory.

The theory of competitive advantage emphasizes the key role of innovation in a country’s competitive advantage (Porter, 1991). Competitive advantage is closely related to artificially created resources, so its position in the international division of labor is dynamic (Feng et al., 2022). In the international division of labor, we need to transform comparative advantage into a competitive advantage to form a real economic advantage (Shao and Li, 2013). The competitiveness of an agricultural product is obtained first through the unique natural geographical environment for its growth and the rich agricultural resources in the region. Due to the natural geographical environment and agricultural resources that feature difficulty to copy or imitate, the regional brand of agricultural products has competitiveness formed by comparative advantage rather than a competitive advantage. This comparative advantage is difficult to sustain. Only by transforming the comparative advantage of regional brands of agricultural products into a competitive advantage can the competitiveness of regional brands of agricultural products be continuously enhanced. The main purpose of this paper is to explore the path that can transform the regional brand’s comparative advantage of agricultural products into a competitive advantage.

#### Research on brand competitiveness of agricultural products

The theoretical research and practical exploration of agricultural product brands outside of China started earlier. Quite a few researchers voiced their own opinions on agricultural product brands from multiple perspectives. Takahashi (1999) pointed out from a cognitive standpoint that agricultural product brands can ensure the quality of food and increase the added value of products so that consumers are more willing to buy products with certain brand awareness. Clemens (2002) investigated the market situation of onion in Iowa, finding that the consumption concept of customers endows the international brand of Vidalia Onion with a high added value, and he proposed that to realize this added value in the marketing chain of agricultural products, the name, quality and image of agricultural products must be protected through trademark ownership. Hardesty (2005), who approached the issue from the financial level, found that the profits of 40 large agricultural cooperatives are as high as US $57.4 billion, but few of them had high brand awareness and reputation. Harbor et al. (2006), who analyzed the issue concerned from the behavioral level, put forward the conceptual model of brand loyalty and believed that the impact of customer brand loyalty on agricultural product brands should be improved from four aspects: customer attributes, customer attitude, product attributes, and media communication.

Other scholars put forward their points of view on the aspects of brand creation, quality certification, brand management, and government support. In terms of the brand creation of agricultural products, Hayes et al. (2003) noted that individual farmers could not bear the expenses and costs incurred by “creating” and “maintaining” brands that can be recognized by customers and are difficult to imitate, for they do not possess the bargaining power and have a small scale of output. Therefore, farmers must create their brands and obtain reasonable profits by limiting product supply. In terms of quality certification of agricultural product brands, Gonzblez-Diaz et al. (2003) pointed out that quality plays an important role in the management of agricultural products, based on their study of the Spanish fresh meat brands. Then they put forward the working procedures for organizing the product quality certification and establishing various organizational forms according to specialized brands. Finally, they proposed the solution to the information asymmetry in the management of quality mechanisms through brand specialization. In terms of brand management of agricultural products, Innes and Kerr (2007) explored ways to improve the competitiveness of agricultural products export in the international market, proposing that, to manage the brand of agricultural products, we must strengthen the management of national image and establish a good national brand image by serializing multiple agricultural products under the same brand, and pay attention to product optimization and quality assurance mechanism to improve the competitive advantage of national agricultural product brands in the international market. In terms of government support for agricultural product brands, Bureau and Valceschini (2003) pointed out that the EU has strengthened the competitiveness of agricultural products in the region through the food labeling policy, successfully promoted product differentiation, and rendered 572 agricultural products to be protected by the EU policy. It has strengthened the management of the place of origin of agricultural products, increased agricultural income, and reduced costs accordingly. Unfortunately, official corruption in the food labeling policy exists, lack of national recognition for products, low product quality, etc. When studying the competitive strategies adopted by individual farmers, Swanson (2003) and Atkins (2005) found that individual farmers cannot adopt product differentiation and marketing strategies like private enterprises, which causes individual farmers to compete directly with other farmers for almost homogeneous products. In addition, the output of individual farmers is limited and cannot meet the needs of many retailers. Finally, considering the high cost of organizing individual farmers, Swanson pointed out that for a long time, government departments have affected the marketing of international agricultural products, so they may help overcome these problems of agricultural products.

Though they started a bit later, Chinese scholars have made achievements on agricultural product brands as follows:

In terms of the necessity and inevitability of brand creation of agricultural products, Wang and Jia (1999) pointed out that, like the marketing of other industrial products, the marketing of agricultural products also involves the creation of the brand, so the brand strategy can be implemented for agricultural products. The government should be aware that the agricultural product brand may have a huge effect. Wang (2007) proposed in his research on the brand development strategy of advantageous agricultural products in Hunan that agricultural branding, especially the brand development of advantageous agricultural products, plays an important role in driving the development of the regional economy. In terms of the relationship between agricultural product brand and quality certification, Kong (1999) argued that product quality must be attached to the brand management of agricultural products and that brand management must be implemented in developing quality agriculture. Cao and Zeng (2000) believed that it is imperative to develop green agricultural products in the brand management of agricultural products. Xu and Du (2002) put forward the research on the brand management strategy of China’s agricultural products under the framework of the WTO. Gan and Li (2003) created the new concept of regional brand agriculture and noted that regional brand agriculture is a new way to accelerate the development of China’s agriculture under the new situation.

In terms of the research on the specific problems and practical experience concerning agricultural product brands, Chinese researchers have employed different methods to explore the competitiveness of agricultural product brands from different perspectives and in combination with local and industrial characteristics. Li and Zeng (2005) conducted an in-depth study on the international competitiveness of Hunan rice. Taking “Yuandong” White Peach, a brand in the hometown of Shi Guangnan, a famous musician in China, as an example, Fang (2003) found that the brand popularity and reputation of agricultural products can be promoted by improving the quality of agricultural products and excavating the cultural heritage, which will, in turn, drive the development of a regional economy. Zhang (2004) pointed out that because of the huge competitive pressure and the weak competitiveness of agriculture in the western region of China, the core of improving the competitiveness of agriculture in the western region lies in enhancing the BC of agricultural products. Wang and Yao (2007) analyzed the factors affecting the BC of agricultural products by employing the Principal Component Analysis, thus quantifying the BC model of agricultural products on this basis.

Previous studies in China and overseas mostly explored BC’s meaning from a brand essence. Few academics empirically analyzed how many variables impact BC of agricultural goods (Baziana and Tzimitra-Kalogianni, 2019; Liu and Wang, 2022). Chinese researchers’ research on regional BC of agricultural goods focuses on ideas, concepts, and assessment factors and lacks empirical evidence, so it cannot provide a practical reference for our empirical investigation. Given the research gap, based on theoretical analysis and previous studies, as well as in-depth interviews with and questionnaires on respondents from the two main productive regions of Jilin ginseng and sika deer, where the author personally took part in the Industrial

Planning Practice of “Changbaishan Ginseng” and “Sika Deer,” this study will construct a theoretical model of the influencing factors for regional BC of characteristic agricultural products, making an empirical study using the Structural Equation Modeling, to explore the relationship between the influencing factors for regional BC of agricultural products, and chart the promotion path of BC of traditional advantageous characteristic agricultural products.

### Conceptual model of regional brand competitiveness of agricultural products

Zhou (2010) and Yao (2013) divided the evaluation indicator system of regional BC of agricultural products into four main levels of criteria: regional factors, brand factors, industrial factors, supporting factors, and 16 levels of sub-criteria. Cai (2010) divided the evaluation indicator system of BC of agricultural products into four levels of criteria levels: enterprise-level, consumer level, social level, government level, and 20 levels of indexes. Shen et al. (2015) divided the evaluation indicator system of BC of industrial clusters into explicit competitiveness and implicit competitiveness, which were further divided into four secondary indicators. Li and Song (2013) and Xu and Wang (2020) identified five latent variables of the structural equation model of regional BC of agricultural products: brand resource capacity, brand foundation capacity, brand support capacity, brand development capacity, and BC capacity. The above research results are rationally viewed from their perspectives.

**FIGURE 1:**
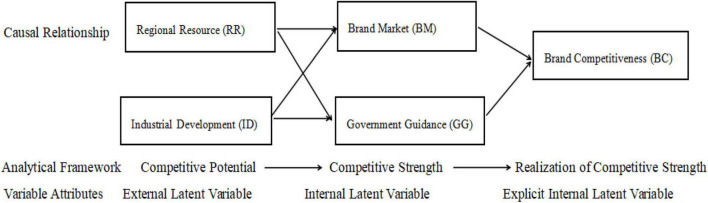
Conceptual model.

Based on the combing of the above literature and the case study of two typical agricultural specialty industries, by taking the Causality Model proposed by Jin (1996) in his *Competitiveness Economics* that involves measuring the competitiveness of industry from the perspective of causes and results, this paper put forward the Conceptual Structural Equation Model of Causality of BC of traditional advantageous characteristic agricultural products (Figure 1). The Causality Model is one of the main methods of a quantitative prediction model, which is mainly used to reveal the correlation between different variables. The Structural Equation Model is a typical causality model.

As revealed in Figure 1, RR capacity and ID capacity are external latent variables, BM capacity and GG capacity are internal latent variables, and BC capacity is an explicit internal latent variable. The above four factors combine to guarantee the realization of regional BC of characteristic agricultural products.

### Hypothesis development

#### Regional resource capacity, brand market capacity, and government guidance capacity

Regional resource capacity is a terminology of the theory of comparative advantage. It reflects the brand effect of the habitat of agricultural products, which feature location endowment, ecological environment, traditional culture, and characteristic folk customs of a specific region. Location resources are the element to promote the agglomeration of industrial clusters of agricultural products, which is derived from the advantages of their characteristic resources (Shang, 2016), thus playing a role in promoting industrial development. The development of industrial clusters and their modernization will also promote the development endowment of characteristic brand resources of the clusters of characteristic agricultural products (Deng and Xiong, 2012). Prahalad and Hamel (1990) found through empirical analysis that the excess profit of enterprises does not come from the characteristics of market structure, but the difference in resource endowment. Derden-Little and Feenstra (2006) studied the regional agricultural marketing plan in California and found that the brand naming of agricultural products based on their places of origin can improve consumers’ cognition and help increase farmers’ income. Koike et al. (2006) measured the influence of regional brand agricultural products such as rice, potato, and milk in Japan from five aspects: brand awareness, brand association, brand image, brand loyalty, and perceived quality of the brand. Taking the “Yuandong White Peach” as an example, Fang (2003) examined ways to improve the brand awareness and reputation of agricultural products by improving the quality of agricultural products and excavating cultural heritage, which will, in turn, drive the development of a regional economy.

The ecological environment of agricultural products can directly affect the behavior of the local government and the growth of the market competitiveness of agricultural products (Li, 2017). Jilin Province of China has issued laws and regulations to protect the woodland for ginseng planting, thus safeguarding the ecological environment of ginseng-producing areas (Li and Sun, 2010). The New Zealand government gives priority to the ecological and environmental protection and standardized production of deer breeding, and the government and the deer associations uniformly manage the international market sales, thus creating conditions for New Zealand’s deer antler and other deer products to seize the international market (Li, 2010; Zheng et al., 2015). The international competition for ginseng and pilose antler begins with planting and breeding and extends to processing, sales, and new product development. The ecological surroundings of product planting and breeding and product processing standards affect agricultural product brands’ popularity and international competitiveness (Long, 2021). Based on the above analysis, we put forward the following hypotheses:

H1: RR capacity has a positive impact on BM capacity.
H2: RR capacity has a positive impact on GG capacity.

#### Industrial development capacity, brand market capacity, and government guidance capacity

Industrial chain theory is used to calculate the ID capacity. In the agricultural industrial chain, several upstream and downstream linkages and value transfers exist. Product or service information flows from the upstream chain to the downstream chain, and the other way around (Siswoyo, 2021). Among the most important components of an ID’s capability is the creation of technological innovation in industrial clusters and the maintenance of industry standards and leading firms (Raiko and Cherepanova, 2019). The implementation of industrial standards means that governments of various countries shall organize the formulation, release, and implementation of standards for all activities of agriculture before, during, and after production, supervise the implementation of agricultural standards, standardize the market order of agricultural products, guide production, and consumption, and achieve the purpose of improving the regional BC of agricultural products, following the requirements for agricultural standardization formulated by the CAC, OIE, and IPPC under the Food and Agriculture Organization of the United Nations (FAO) (Wu, 2011). At present, agricultural products in developed countries have been standardized. Under the guidance of the Chinese government in Jilin has established the industrial standards for industrialized ginseng planting, promoted the standardized and normalized production of ginseng from the source of its plantation, specified the industrial standards for ginseng planting, processing and operation, and comprehensively enhanced the “safe and high-quality ginseng production technology” and “soil testing-based ginseng planting technology,” to realize the rural revitalization through leading industries (Guo, 2019).

According to Gonzblez-Diaz et al. (2003), the quality of agricultural products is the “lifeblood” of regional brands of agricultural products and an important guarantee for the quality and safety system of agricultural products; meanwhile, the quality of agricultural products has won the recognition and favor of consumers in the management of agricultural products. The public attribute of regional brands determines that the government shall participate and play a guiding role in establishing the regional brands of agricultural products. As revealed by von Malmborg (2007), when issues like the quality and safety of agricultural products constantly affect the brand image of the unique regional agricultural products, the agricultural enterprises themselves are not capable of conducting regional brand construction and realizing the integration of local brands, so that the guidance of the government is required. Agricultural product enterprises often pay more attention to the brand registration of agricultural products while ignoring the brand management so that they are often confronted with problems like a small scale of brand produces, scattered operation, and no advantageous position in market competition (Olson, 1968). All these problems require the government to promote the regional brand construction of local agricultural products. The industrial cluster development of the regional agricultural products needs to be placed under the organization and management of the government, industry associations, and enterprises to promote the construction of the industrial value chain system. Based on the above analysis, the following hypotheses are put forward:

H3: ID capacity has a positive impact on BM capacity.
H4: ID capacity has a positive impact on GG capacity.

#### Brand market capacity and brand competitiveness capacity

Brand market capacity reflects both the performance of comparative advantages and the value of brand equity exhibited by brands in market competition. Brand equity is an important part of BC. According to Aaker (1991), brand equity comprises brand awareness, perceived quality, brand associations, brand loyalty, and other exclusive equities of the brand. Keller (1993) put forward the Consumer-Based Brand Equity (CBBE) model and proposed that the six elements that characterize a successful brand are brand salience, brand performance, brand imagery, consumer judgments, consumer feelings, and brand resonance. These six elements are placed onto four levels: identity, meaning, response and management. Brand building should be carried out on these four levels. As is pointed out by Lassar et al. (1995), brand trust connotes a sense of security that customers get from the brand, and the brand can meet customers’ expectations. Crosby et al. (1990) believed that brand trust is gradually formed with the continuous information acquisition and accumulation of both customers and brands. It is a rational cognitive process and behavior. Trust will lead to customers’ recognition of the corporate brand. Jacoby and Chestnut (1978) proposed that brand loyalty is the ratio of the number of times consumers buy a brand against the number of times they buy all products of that category. The higher the percentage, the greater the brand loyalty. Bennett (2002) deemed that brand loyalty is an attitude of the customers–their preference for and psychological commitment to a specific brand.

Brand competitiveness capacity represents both the external market expressiveness that highlights the comparative advantage of a specific enterprise over its competitors and the degree of recognition of and preference for the brand exhibited by the customers in the process of brand consumption. It is the market forces generated by the above two factors in the market activities of the brand. Shen et al. (2005) research revealed that the BC indicator system comprises both explicit and potential indicators. Explicit indicators mainly include market share, market coverage, and sales profit margin. The Interbrand Group of Britain estimates the BC mainly according to the enterprise’s market share, product sales, and profit status, combined with the subjective judgment of brand strength (Stobart, 1994).

The regional brand of agricultural products is the condensation and crystallization of the quality and quantity of agricultural products. It symbolizes specific attributes and represents the high quality of agricultural products (Liu and Wang, 2022). It is a reliable sign that communicates the quality and safety of agricultural products to consumers, helping to increase brand awareness and trust. These are all conducive to improving agricultural products’ added value and BC value.

Based on the above analysis, the following hypotheses are put forward:

H5: BM capacity has a positive impact on BC capacity.

#### Government guidance capacity and brand competitiveness capacity

Government guidance refers to the thorough management of regional trademarks of distinctive agricultural goods carried out by entities such as the government, trade associations, businesses, and so forth within the region. It consists of high-level design and industrial planning, policy and regulation guidance, industry association oversight, and a complete service system (Perskaya et al., 2020). Research by Zhang and Liu (2015) showed that the public attribute of the regional brands of agricultural products objectively determines that the government should participate in and take the leading role in brand creation. The promotion of the regional brands of agricultural products consists in establishing a good institutional environment by the government, as well as promoting the continuous deepening of the division of labor, the strengthening of cooperation and innovation among them, and the economic synergy among different enterprises, thus boosting the concordant evolution of industrial clusters. Based on a study of the government behavior in the brand integration of “Changbaishan Ginseng,” Wang and Li (2014) found that when enterprises and farmers themselves cannot complete the shift from scattered original planting to industrialization development of ginseng in the region, the government must intervene and play the guiding role to increase farmers’ income effectively.

According to Yang and Liu (2017), the relevant entities of regional brands provide sound organizational guarantees for the brand operation of characteristic agricultural products through policy support and organizational coordination. Sun (2009) demonstrated that the generation mode of industrial clusters and regional brands would determine the role and function of local governments. If the constructiveness of the government is greater than the spontaneity of the market, then the government will play a leading and influencing role, and the construction of regional brands will be guided by government behavior. Zhang (2009) proposed that the government should be the formulator and maintainer of the quality standardization system of brand agricultural products. Through a case study on the garlic industry in Jinxiang County, Shandong Province, Zheng and Gu (2006) showed that the government should play a greater role in basic industrial platforms, regional brands, regional marketing, and industrial upgrading. Kotval and Mullin (1998) deemed that it is a necessity to establish a novel and influential industry association in the industrial cluster. As was pointed out by Rosenfeld (2003), the implementation of cluster strategy often starts with the establishment or approval of an industry association, whose role is to carry out activities on behalf of all enterprises of the cluster and to meet all needs. The GG will build up the positive role of RRs and ID in promoting the construction of regional BC. Based on the above analysis, the following hypotheses are put forward:

H6: GG capacity has a positive impact on the BC capacity of agricultural products (BC).

#### The mediating role of brand market capacity

The BM capacity plays a mediating role between RR capacity, ID capacity, and regional BC. According to research by Gonzblez-Diaz et al. (2003), conducting production management according to standards, improving the quality of agricultural products, and ensuring the safety of agricultural products are the most effective measures and means to improving customer satisfaction and customer trust in the brand, which also becomes the cornerstone for building BC. As was pointed out by Thomas (1998), the development of new products by enterprises with the help of technological innovation capacity will play the role of improving enterprise brand image, obtaining more benefits for enterprises, and winning competitive advantage for them. Zeithaml et al. (1996) revealed that brand loyalty represents the customers’ attempt to maintain a relationship with the target brand through a series of behaviors, mainly including the dissemination of word of mouth and repeat purchases, which will enhance the market competitiveness of the brand. Customer loyalty is the key to the formation of BM’s competitiveness and promotes online shopping of various products (Wattoo and Iqbal, 2022). To establish a solid foundation of BM capacity, we must win considerable loyalty from the consumers. Cultivating customer loyalty is the fundamental way to improve the BM capacity. The improvement of customer loyalty can improve the BM capacity.

Based on the above analysis, the following hypotheses are put forward:

H7: BM capacity plays a mediating role between RR capacity and BC capacity.
H8: BM capacity plays a mediating role between ID capacity and BC capacity.

#### The mediating role of government guidance capacity

Government guidance capacity is a notion formed from the theory of brand equity and the public qualities of regional brands. Based on brand equity theory, the government can use the brand to encourage the growth of the local economy (Davcik et al., 2015). So that the agricultural brand enterprises will pay more taxes to the government while winning more income and profits for themselves. The GG may also improve the local employment, escalate the construction of the production base, and promote industrial optimization. This motivation, which conforms to the government’s requirement for brand equity, will motivate the government to support and safeguard the regional brand creation of agricultural products and promote the formation of regional BC agricultural product capacity (Liao et al., 2021). According to Zhou and Wang (2008), driven by the brand construction of the government, local agricultural resources may win comparative advantages, to expand the regional influence of their brands, enabling more people better to understand the local cultural features through agricultural product brands, to enhance their trust in the government, and to finally drive the rapid development of other local industries. Rosenfeld (2003) pointed out that as a governance mechanism, industry associations can not only coordinate and standardize industries and enhance the self-discipline of industries, but also provide enterprises with more information and reduce the occurrence of information asymmetry in market transactions. The associations are a coordinator between the government and enterprises, seeking government help or policy support for enterprises while representing members of the cluster and carrying out activities that will meet the needs of all enterprises in the cluster. The trade associations play an active role in promoting the development of regional brands of agricultural products, enhancing the cooperation of enterprises in the fierce market competition, escalating the construction of industrial chain and value chain system, and finally raising the competitiveness capacity of regional brands.

We proposed that brand management can actively and effectively mediate RR and ID’s mediating role in regional brand construction. In addition, viewed from the Structural Equation Measurement model, there might be some correlation between the two external latent variables of RR and ID, which needs to be tested here. Based on the above analysis, the following hypotheses are put forward:

H9: GG capacity plays a mediating role between RR capacity and BC capacity.
H10: GG capacity plays a mediating role between ID capacity and BC capacity.
H11: There is a positive correlation between RR capacity and ID capacity.

## Materials and methods

**FIGURE 2:**
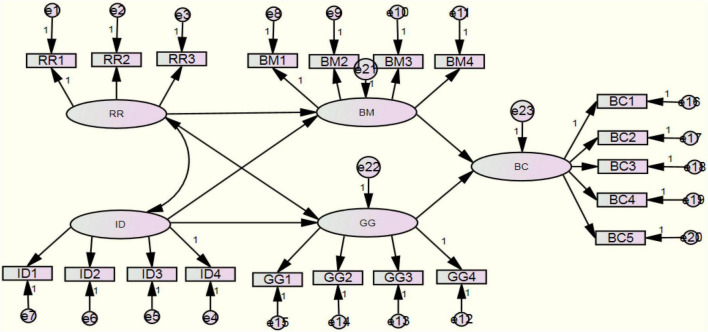
Structural equation modeling (SEM) model of a causal relationship in regional BC of traditional advantageous characteristic agricultural products.

The main statistical tools employed in this study are Structural Equation Modeling (SEM) and the Bootstrap method. The SEM and Bootstrap methods are superior to average methods in testing the relationship between latent variables. SEM has two built-in models: The measurement Model and Structure Model. The former includes latent variables and observation variables, while the structural model explains the causal relationship between latent variables (Wu, 2007). SEM is a verifiable modeling method, which can conduct an ideal theoretical test of hypotheses based on key theories and concepts. According to the conceptual model of causality for the regional BC of traditional advantageous characteristic agricultural products (Figure 1), we established a Measurement model (Figure 2) to demonstrate the hypothetical path.

The SEM and Bootstrap method are employed in this study to test the relationship between potential variables, to verify the mediating effects of two mediating variables: BM capacity and GG capacity: that is, the mediating role of BM capacity between RR capacity, ID capacity and BC capacity and the mediating role of government support capacity between RR capacity, ID, and BC. The Bootstrap method is used to test whether the mediating effect exists. In addition, it is also conducive to the analysis of complex mediating effects, such as the adjustment of mediating variables and the coordination of multiple mediating effects. Our research is aimed at analyzing two coordinated mediating variables, so the Bootstrap method becomes the most appropriate and preferred method.

### Variable selection

#### Regional resource capacity and industrial development capacity

The RR capacity reflects the unique and irreplaceable attributes of characteristic agricultural products, formulates the comparative and differential advantages of regional agricultural products compared with others, and forms the basis of BC of characteristic agricultural products. As the core of the BC of characteristic agricultural products, the ID capacity reflects the quality of characteristic agricultural products, including various elements of the whole industrial chain of agricultural products. The above two capacities combine to lay a foundation for the BC of characteristic agricultural products, forming the two external latent variables of the Structural Equation Model of BC of characteristic agricultural products. Based on the research results of Yao (2013), we adopted the three observation variables of a brand’s natural resources, ecological resources, and historical and cultural resources to evaluate the RR capacity. Then based on the research results of Cai (2010); Zhou (2010), Wu (2011), and Zhang and Liu (2015), the four observation variables of industrial standards, agricultural enterprises, product quality, and technological innovation are adopted to evaluate the ID capacity. As external latent variables, RR capacity and ID capacity may form a covariant relationship between them, which needs to be verified in the test.

#### Brand market capacity and government guidance capacity

The BM capacity reflects the interaction between brands and consumers, the recognition and satisfaction of consumers with the regional brands of characteristic agricultural products, determining consumers’ willingness and behavior in choosing similar agricultural products, and the market influence of the characteristic agricultural brands. Based on the research results of Xu (2004) and Koike et al. (2006), we selected four observation variables: brand awareness, brand satisfaction, brand reputation, and brand loyalty to evaluate the BM capacity.

The GG capacity reflects the interaction between brands and managers, and the joint construction of regional brands by the stakeholders of industrial clusters through the co-construction of the mediating platform and competitive-cooperative mechanism of brand co-creation. The GG is the key to the regional brand construction of characteristic agricultural products, which determines the orientation of the industry of characteristic agricultural products and the prospect of BC. Based on the research results of Zhang (2015) and Xu and Wang (2020), we evaluated the GG capacity with four observation variables: top-level design and planning, policy and financial support, industry association supervision, and service system construction.

#### Brand competitiveness capacity

The BC capacity refers to the market influence of regional brands of characteristic agricultural products, including the market’s external performance and sustainable development. Based on the research results of Zhang (1996) and Shen et al. (2015), this paper evaluated the BC with five observation variables: market share, sales profit margin, market coverage, market sales, and foreign exchange earned through export (exchange rate appreciation).

### Questionnaire survey and data collection

#### Design of in-depth interview and initial questionnaire

The questionnaire items of this study are fixed based on data from in-depth field work interviews, as well as the measurement scale and related theoretical framework of previous scholars on the influencing factors of regional BC of agricultural products. The 5-point Likert scale is adopted to assign the numerical values of 1–5 to the influence degree of each factor, which, respectively, represents “strongly disagree,” “disagree,” “basically agree,” “agree,” and “strongly agree.” See Table 1 for five latent variables and 20 observation variables and their sources of the structural equation measurement model.

**TABLE 1:** Measured items and their sources.

Latent variable	Observation variable	Source of the observation variable
RR	(1) Location endowment (RR1)	Yao, 2013
	(2) Ecological environment (RR2)	Yao, 2013
	(3) Historical and cultural resources (RR3)	Yao, 2013
ID	(1) Industrial standard (ID1)	Zhang and Liu, 2015
	(2) Leading enterprise (ID2)	Zhou, 2010
	(3) Product quality (ID3)	Zhang and Liu, 2015
	(4) Technological innovation (ID4)	Cai, 2010
BM	(1) Brand awareness (BM1)	Xu, 2004
	(2) Brand satisfaction (BM2)	Xu, 2004; Koike et al., 2006
	(3) Brand reputation (BM3)	Xu, 2004; Koike et al., 2006
	(4) Brand loyalty (BM4)	Xu, 2004; Koike et al., 2006
GG	(1) Development strategy and planning (GG1)	Zhang and Liu, 2015
	(2) Policy support and guarantee (GG2)	Xu and Wang, 2020
	(3) Online marketing promotion (GG3)	Xu and Wang, 2020
	(4) Service system improvement (GG4)	Zhang and Liu, 2015
BC	(1) Market share (BC1)	Zhang, 1996; Shen et al., 2015
	(2) Sales profit margin (BC2)	Zhang, 1996; Shen et al., 2015
	(3) Market coverage (BC3)	Shen et al., 2015
	(4) Market sales (BC4)	Shen et al., 2015
	(5) Exchange rate appreciation (BC5)	Shen et al., 2015

The initial design of the questionnaire items is based on the interviews, inquiry, and argumentation at a meeting convened by the local governments and attended by heads of the leading enterprises and industry associations of some regional ginseng and sika deer enterprises, as well as related government officials, experts, and scholars, when the author was participating in the industrial planning of China’s Jilin WL ginseng and Jilin SY Sika Deer from October 2018 to 2019. The interviews and discussions add up to 41 persons, among them, there are 27 people from the ginseng industry, accounting for 65.85%: 14 people from the sika deer industry, accounting for 34.15%. The author also visited local ginseng and sika deer enterprises, ginseng growers, sika deer farmers, and marketing personnel. The first draft of the questionnaire was formulated by combing and sorting out the accumulated data.

The first draft of the questionnaire was mailed to the heads of several ginseng and sika deer enterprises, related governmental officials, industry associations, and college and university experts concerned for suggested modifications. The questionnaire was revised several times according to the feedback opinions, and the initial questionnaire of this survey was finally formulated, including the basic information of respondents such as gender, age, and education, as well as 23 questions comprising the main part. A total of 160 initial questionnaires were sent via the Internet and mobile WeChat, of which 125 were returned, accounting for 78%. 102 of these questionnaires are valid, accounting for 63.75%.

#### Formation of the formal questionnaire

After the CITC, reliability and validity analysis of the initial questionnaire items, 20 items of the initial questionnaire passed the verification of reliability and validity, thus forming a formal questionnaire. Subsequently, a questionnaire survey was conducted in the main production areas of ginseng and sika deer in Jilin, China, and sample data were collected. 289 questionnaires were finally recovered, of which 75 unqualified questionnaires were excluded for reasons like lack of understanding of the characteristic agricultural brands, option mission, and regular tick of options. The valid questionnaires finally recovered added up to 214, accounting for an effective recovery rate of 74.05%. Among them, 125 questionnaires involve the ginseng industry, accounting for 58.41%; 89 questionnaires involve the sika deer industry, accounting for 41.59%; the number of males is 129 males, accounting for 60.28%; the number of females is 85, accounting for 39.72%. The percentages of respondents’ age and education are shown in Table 2.

**TABLE 2:** Description of demographic.

Sample attribute	Category	Sample number	Percentage (%)
Category of respondents	Ginseng industry	125	58.41
	Kita deer industry	89	41.59
Gender of respondents	Male	129	60.28
	Female	85	39.72
Age of respondents	Below 30	41	19.16
	31–40	95	44.39
	41–50	59	27.57
	Above 50	19	8.88
Education of respondents	Below high school	27	12.62
	High school	63	29.44
	Two- or 3-year college	70	32.71
	Undergraduate	42	19.63
	Postgraduate	12	5.61
Total		214	100

SPSS v22 and AMOSS v18 were used to analyze the data. The testing process of the model is as follows: First, we tested the common method biases by the Harman test, conducted a goodness of fit analysis on the external quality of the overall model through a series of models, and evaluated the internal quality of the model through reliability, validity test, descriptive statistics, and Pearson correlation. Then, we used the SEM path analysis to test the hypotheses. Finally, we used the Bootstrap method to test the mediating effects of mediating variables and the different mediating roles between the mediating variables in different paths.

## Results

### Common method deviation

The common method biases and possible social desirability bias are mostly related to the same variable data sources. This study used the program control method and statistical test method to control the possible deviation of general methods. Specific methods include: (1) We emphasize that the purpose of our questionnaire survey is merely for academic research, we do not obtain any information concerning trade secrets, and there are no correct or wrong answers to questions on the anonymous questionnaire; (2) The questionnaire items were so designed as to avoid too high or too low social desirability of a certain attribute; (3) We used the Harman univariate test to load the observation variables of all latent variables into a single factor model, where the proportion of variance explained by the first factor accounts for 39.875% (less than 40%), indicating that the common method deviation is within the allowable range (Sun and Li, 2017).

### Model fit analysis

The R-squared evaluation of the Structural Equation Model was mainly based on three groups of indices: absolute fit indices, incremental fit indices, and simple fit indices, and a comprehensive evaluation was made by requiring most indices to meet the standards (Hair et al., 1998). In this study, the maximum likelihood estimation was used for model calculation, and the final result obtained through several modifications is χ^2^ = 286.704, indicating that the goodness of fit of the model matches well; and χ2/df = 1.759, indicating a very good fitting effect of the model. The RMSEA value is 0.060, indicating that the goodness of fit of the model matches well; the GFI value is 0.884 and the AGFI value is 0.850, indicating that the absolute fit indices meet the recommended standards. The incremental fit index CFI is 0.94, the IFI is 0.94, and the NFI is 0.872, all of which are greater than 0.80, meeting the recommended standards for incremental fit indices. The simply fit index values PGFI and PNFI exceed the recommended standard of 0.50, showing a good fitting effect (see Table 3).

**TABLE 3:** Evaluation of the fitting degree of the measurement model.

Type	Absolute fit indices					Incremental fit indices			Simple fit indices	
Goodness of fit	*X* ^2^	*X*^2^/df	RMSEA	GFI	AGFI	CFI	IFI	NFI	PGFI	PNFI
Statistical value	286.704	1.759	0.06	0.884	0.85	0.94	0.94	0.872	0.686	0.748

### Reliability and validity analysis

The R-squared test of the overall model cannot reflect the internal quality of the model. Therefore, we conducted key reliability and validity tests to determine whether the applicability of our measurement model meets our research purpose. We used SPSS22.0 to evaluate the composite reliability and construct validity of the model.

The internal consistency reliability was tested through the coefficient of “Cronbach’s α.” Based on the viewpoints of DeVellis (1991) (as cited in Wu, 2010, p. 244), this paper took 0.6 as the acceptable standard for Cronbach’s coefficient in the exploratory research. In terms of reliability, we used the corrected item-total correlation (CITC), where a greater than 0.3 value means passing the test according to the suggestions made by Lu et al. (2007). We adopted the value of 0.4 by the practice of most previous studies. The Kaiser Meyer Olkin (KMO) test was carried out to determine whether the factor analysis is suitable between items. According to Kaiser (1974), we adopted the greater than 0.60 value as the criterion for judgment. Bartlett’s test of sphericity is significant at the level of 0.01. Thus, judgment was made on whether the data are suitable for the exploratory factor analysis (EFA).

(1) The CITC and reliability test of the measurement items for each latent variable. In the item analysis, we deleted the following items: the item whose correlation coefficient with the total score is less than 0.4 and not significant (note: the significant value of *P* < 0.01); the item the deletion of which would cause the corresponding internal consistency (α coefficient) to increase. According to the analysis results, 3 items whose correlation coefficient is less than 0.3 were deleted and 20 items were retained. The Cronbach’s α value of each latent variable of the revisedquestionnaire is 0.812, 0.829, 0.844, 0.825, and 0.859, respectively, which is all greater than 0.6; the “Cronbach’s α value after deleting this item” of each measurement item below the latent variables of the questionnaire is lower than the “Cronbach’s α value of each variable before the item was deleted,” where the coefficient values between the item and total score are greater than 0.4 and significant at the level of 0.01, indicating that the measurement items of each variable have high internal consistency. Therefore, the remained 20 measurement items all passed the CITC and reliability test.

(2) The CITC and reliability test of the measurement items of the whole questionnaire. Test results show that as concerns the overall questionnaire on the BC of traditional advantageous characteristic agricultural industries, Cronbach’s α = 0.920 and the results based on standardized terms are Cronbach’s α = 0.920. The Cronbach’s α value of each item after deleting this item is less than 0.886. The correlation coefficient of the measurement item-total score of each measurement variable is greater than 0.4 and significant at the level of 0.01, indicating that each measurement item below the potential variables of the whole questionnaire has high internal consistency, and the measurement items of all potential variables passed the CITC test.

(3) The validity analysis of each latent variable. In this paper, factor analysis was adopted to test the construct validity of the data. SPS22.0 was used to analyze each latent variable, showing that the KMO values of RR, ID, BM, GG, and BC are 0.713, 0.800, 0.805, 0.731, and 0.840, respectively, which are all greater than 0.6, and the significance level of Bartlett test of sphericity results is averaged at 0.000 and less than 0.001. The total cumulative variance explained by each latent variable is more than 50% (Wu, 2010), indicating that the latent variables have good construct validity and the data results are suitable for factor analysis (see Table 4).

**TABLE 4:** Test results of model reliability and validity.

Variable	Cronbach’s α	Items	CITC	KMO value	Bartlett *P*-value	Loadings	Cumulative variance (%)
RR	0.812	RR1	0.685	0.713	0.00	0.867	72.853
		RR2	0.636			0.858	
		RR3	0.669			0.836	
ID	0.829	ID1	0.677	0.8	0.00	0.860	66.579
		ID2	0.724			0.831	
		ID3	0.64			0.804	
		ID4	0.594			0.765	
BM	0.844	BM1	0.642	0.805	0.00	0.848	68.192
		BM2	0.689			0.834	
		BM3	0.71			0.824	
		BM4	0.677			0.797	
GG	0.825	GS1	0.633	0.731	0.00	0.819	65.632
		GS2	0.647			0.816	
		GS3	0.659			0.807	
		GS4	0.662			0.798	
BC	0.859	BC1	0.68	0.84	0.00	0.839	64.032
		BC2	0.64			0.813	
		BC3	0.637			0.805	
		BC4	0.726			0.772	
		BC5	0.695			0.770	

(4) The validity analysis of the total questionnaire. Analysis of the whole questionnaire on the BC of characteristic agricultural industries shows that the KMO value of the whole questionnaire is 0.900, which attains the “Good” standard and is suitable for factor analysis. By the maximum variance method for factor analysis, the factor load of most measurement items of each latent variable is higher than 0.5. Five common factors were automatically extracted by orthogonal rotation, and the cumulative variance explained is 68.539%, which is larger than 50%, indicating that the total questionnaire has good construct validity.

### Descriptive statistics and correlation analysis

The correlation analysis must be done before hypothesis testing. From the descriptive statistics in Table 5, we found that the mean of all latent variables is greater than the standard deviation, which indicates that the measures of dispersion of all data are moderate so that subsequent correlation analysis could be carried out. According to Pearson, in inferential statistics, we cannot judge whether the correlation between two variables is significant or not merely from the absolute value of the correlation coefficient, for the judgment must be made from the value *p* of the significance test on the correlation coefficient. If the significance probability is *p* < 0.05, it means that the correlation between two variables is significant. In this paper, the *p*-values of the significance test on the correlation coefficient between model variables are all less than 0.01 (marked by ^**^), indicating that the correlation between variables is significant. From the Pearson correlation coefficient, we found that there were significant positive correlations in varying degrees between all explanatory variables and explained variables. For example, the correlation coefficient of RR and ID is 0.372, indicating that there is a significant positive correlation between these two variables, and there is also a significant positive correlation between other variables.

**TABLE 5:** Descriptive and correlation.

Variable	Array average	Array st. d.	RR	ID	MC	GG	BC
RR	3.774	0.6559	1				
ID	3.572	0.6873	0.372**	1			
BM	3.732	0.6696	0.428**	0.546**	1		
GG	3.627	0.6615	0.506**	0.507**	0.462**	1	
BC	3.777	0.6982	0.453**	0.489**	0.576**	0.493**	1

**Indicates a significance level of 1% (two-tailed).

Therefore, we can conclude that such data can be used as the basis of a hypothesis test.

### Structural equation modeling analysis of direct impact

**FIGURE 3:**
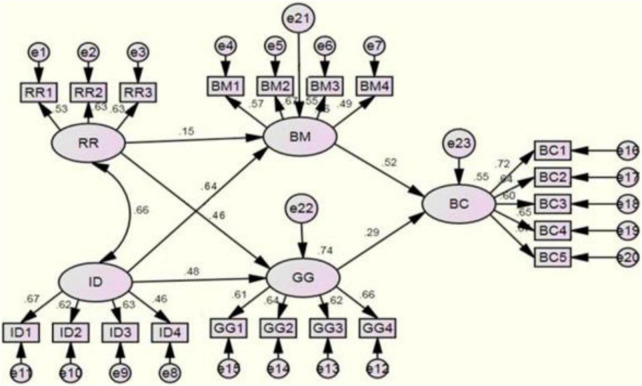
Path model.

Structural equation modeling was applied to test the hypotheses of this study. Figure 3 and Table 6 show that RR has a significant positive impact on BM (whose standard path coefficient is 0.146), thus verifying H1.

**TABLE 6:** Path coefficient of structural equation modeling (SEM).

Path standard	Path coefficient	*SE*	*T*	*P*
RR → BM	0.303	0.081	3.789	***
RR → GG	0.426	0.094	5.171	***
ID → BM	0.523	0.089	5.643	***
ID → GG	0.451	0.092	5.272	***
BM → BC	0.525	0.101	5.775	***
GG → BC	0.317	0.080	3.897	***

***Indicates that *p* < 0.001. SE is the standard error.

RR has a significant positive impact on GG (whose standard path coefficient is 0.462), thus supporting H2.

ID has a direct and important impact on BM. The standardized path coefficient between ID and BM is 0.643. H3 is thus passed.

The standardized path coefficient of the impact of ID on GG is 0.484, thus verifying H4.

Our results reveal the positive and significant impact of BM on BC, and the standardized path coefficient is 0.520. Among all the factors affecting the BC, the influence coefficient of BM is the highest, thus validating H5.

Our study also shows that the GG has a significant positive impact on BC, and the standardized path coefficient here is 0.294. H6 is thus verified.

### Mediating test method

The mediating effect model can analyze the process and mechanism of the influence of independent variables on dependent variables. At present, it is generally believed that the Bootstrap method is better in directly testing the significance of the coefficient product (e.g., Zhao et al., 2010), and the Structural Equation Model is the most suitable tool for analysis of the mediating effect.

The mediating test is aimed at testing the mediating role of the two mediating variables of BM and GG between the two independent variables of RR and ID and the dependent variable of BC. The possible mediating effect of the two mediating variables can be determined by using the Bootstrap method to determine the existence of the mediating effect and the authenticity of corresponding assumptions. We use the Bootstrap method in AMOS and the Process plug-in of SPSS 22.0 to analyze the mediation effect. We set the number of random repeated sampling as 1,000, and the confidence interval as 95%. If the confidence interval does not contain 0, the mediation effect is significant. The specific test steps and results are as follows:

#### The mediating role of brand market/government guidance between regional resource and brand competitiveness

First, we directly test the mediating effect of two mediating variables. The results in Table 7 show that the total mediating effect of BM between RR and BC is 0.294, and the confidence interval is (0.1885, 0.4341), excluding 0, indicating that the mediating effect is positive and significant. In addition, the mediating effects of the two mediating variables were tested, respectively. The mediating effect of BM is 0.159, the confidence interval is (0.1189, 0.3175), the mediating effect of GG is 0.135, and the confidence interval is (0.0989, 0.2927), both excluding 0, both of which are significant positive mediating effects.

**TABLE 7:** Mediating role of brand market (BM)/government guidance (GG) between regional resource (RR) and brand competitiveness (BC).

Indirect effects of BM/GG between RR and BC					
Constructs	Effect	BootSE	BootLLCI		BootULCI
Total	0.294	0.0614	0.1885		0.4341
BM	0.159	0.0510	0.1189		0.3175
GG	0.135	0.0499	0.0989		0.2927

**Direct impact of RR on BC**					
**Effect**	***SE***	***T***	***P***	**LLCI**	**ULCI**

0.4824	0.0652	7.4013	0.000	0.3539	0.6108

We continued to test the direct impact of RR on BC. After controlling the mediating variables, the direct influence coefficient of RR on BC is 0.4824, and the confidence interval is (0.3539, 0.6108), excluding 0, indicating that the mediating effect is positive and significant. The results of this study validate the mediating effect of BM, thus accepted H7. Also, along the path of RR → GG → BC, the mediating effect of GG is also proved, thus validating H8. Because the direct impact is also significant, we can assume the partial mediation effect of selected mediating factors.

#### The mediating role

As mentioned above, following the test steps for mediating effects, we tested the mediating role of BM/GG between ID and BC. The test results are shown in Table 8. Therefore, we concluded that the total mediation effect of BM/GG between ID and BC is 0.417, and the confidence interval is (0.2067, 0.4862), excluding 0, indicating that the mediation effect is positive and significant. The mediating effects of BM and GG were tested, respectively. The mediating effect of BM between ID and BC is 0.275 and the confidence interval is (0.1421, 0.3752); the mediation effect of GG between ID and BC is 0.142, and the confidence interval is (0.0682, 0.2722), excluding 0, indicating that the mediation effect is positive and significant.

**TABLE 8:** Mediating role of BM/GG between ID and BC.

Indirect effects of BM/GG between ID and BC					
Constructs	Effect	BootSE	BootLLCI		BootULCI
Total	0.417	0.0711	0.2067		0.4862
BM	0.275	0.0591	0.1421		0.3752
GG	0.142	0.0523	0.0682		0.2722

**Direct impact of ID on BC**					

**Effect**	**BootSE**	***T***	***P***	**LLCI**	**ULCI**

0.4965	0.0609	8.1571	0.000	0.3765	0.6165

We continued to test the direct impact of ID on BC. After controlling the mediating variables, the impact of ID on BC is still significant, with a direct impact coefficient of 0.4965 and a confidence interval of (0.3765, 0.6165). Along the path of ID → BM → (BC), and the path of ID → GG → BC, the mediating effect of BM and GG is also proved in this study. Therefore, H9 and H10 are statistically accepted.

## Discussion

### Impact of regional resource on brand market and government guidance

The finding is consistent with that of Zhang and Liu (2015), indicating that for the agricultural industry whose product quality is closely related to environmental factors like water, soil, climate, and cultural characteristics of its place of origin, the existence of resource endowment lays the foundation for the growth of characteristic agricultural industries, as well as for the core value of regional brands, which plays a strong driving role in the formation of BM capacity. The result is consistent with the research result of Li and Song (2013), showing that the existence of resource endowment promotes not only the government’s guidance and support for the development of characteristic agricultural industries but also the industry association’s supervision of regional brands of characteristic agricultural products, thus playing a strong role of promotion.

### Impact of industrial development on brand market and government guidance

The research result fully reflects the impact of various factors of the industry chain of the characteristic agricultural products on the quality of brand products, which further obtains the recognition and trust of consumers, rendering them willing to recommend brand products to familiar people, thus improving the BM capacity. The result is consistent with the research conclusion of Xu (2004). The result is consistent with the research conclusion of Shen et al. (2013). That fully demonstrates that the government is an important entity in the development of characteristic agricultural industry and regional brand construction, who offers guidance to the direction and strategy of industrial development, formulate policies and regulations to encourage and support the integration of regional brands, and the industry associations effectively promote the construction of regional brands through the access supervision on and services for regional brands.

### Impact of brand market on brand competitiveness

Meanwhile, the finding is consistent with Wang (2010), who proposed that brand value comes from the totality of the consumers’ brand cognition, that is, consumers’ brand association and brand loyalty, as well as customer, delivered value and brand awareness. To enhance the competitiveness of the brand, enterprises must attach great importance to consumers’ cognition of the brand from the perspective of customer value and improve customers’ loyalty to the brand.

### Impact of government guidance on brand competitiveness

Our research is consistent with the results of Zhang and Liu (2015) that reveal the impact of GG and services on the promotion of regional agricultural brands, showing that the government plays an important role in the construction of regional agricultural brands. However, the government should not act as a direct leader or agent, but it should be a facilitator or promoter. The government should give guidance on the direction of development strategy and planning, formulate promoting and encouraging policies and regulations, and actively offer support to the construction of funds and platforms, thus helping the established industrial clusters for characteristic agricultural products to promote the construction level of the whole industrial chain rapidly, to enhance the market influence and competitiveness of regional brands.

### The mediating role of brand market and government guidance

Our results are consistent with Li and Song (2013) research conclusions. The agricultural resource endowment is the starting point for promoting a regional agricultural brand. The advantage of resource endowment is the basis for improving the BC of a regional agricultural brand. However, we build a mature industrial chain and value chain system in terms of industrial elements, which is the foundation of creating the consumer market. The comparative advantage of resource endowment can truly help realize the BC and form a strong brand only through the synergy of mature industrial elements. Our study verifies the conclusions of Lu et al. (2021). The ID with product quality as the core can enhance consumers’ brand recognition and affect consumers’ brand loyalty, which is consistent with the results of Brown (2005) and Huang et al. (2014). Consumers can fully consider their needs when making purchase decisions and relate them with brand identification. Product quality is regarded as the most basic element of all kinds of information considered by consumers. High-quality products are recognized through consumers’ brand recognition. Once this emotional cognition is generated, consumers can increase the number of purchases and actively publicize the brand, say through word-of-mouth, to help enterprises attract more potential consumers. Brand consumers are becoming ever more loyal to enterprises, which will eventually affect BC.

These studies show that various influencing factors’ influence paths and effects on BC are different. The influencing factors in the descending effect are BM, ID, GG, and RR. The revelation here is that the way to promote the regional BC of traditional advantageous characteristic agricultural products under the background of economic globalization lies with conglomerating the capacities of BM, ID, GG, and RR of the brand to reinforce the regional BC of traditional advantageous characteristic agricultural products. In practice, because different stakeholders of characteristic agricultural industrial clusters have their advantages in different fields, the government should focus on formulating and improving corresponding policies and regulations to form different incentive mechanisms. For brand managers, the government should encourage them to develop the market jointly and vigorously enhance the market influence of the brand; for enterprises and farmers, the government should encourage them to implement quality standards throughout the industrial chain in strict accordance with industry standards, to win the satisfaction and trust of consumers; moreover, strong protection should be given to the RRs and ecological environment of agricultural products.

## Research conclusion and countermeasures

### Research conclusion

Taking the status analysis of the international trade of the two typical regional brands of China’s traditional advantageous characteristic agricultural industries – “Changbaishan Ginseng” and “Jilin Sika Deer” as an example, based on the 214 valid questionnaires from respondents in the two main production areas of WL ginseng and SY sika deer in Jilin, China, this study analyzes with the SPSS 20.0 and AMOS software the relationships between the influencing factors of the regional BC of traditional advantageous characteristic agricultural industries, exploring the mediating role of BM and GG between RR, ID, and BC. Our research results expanded the research frontier in recent years and the main findings are as follows:

(1) BM is the most important factor affecting the regional BC of characteristic agricultural products, which reflects the significance of strengthening BM and points out the direction of improving BC. BM not only has a direct and important impact on BC but also plays an important mediating role between RR, ID, and BC. This reminds us that the brand stakeholders should jointly strengthen market development, jointly promote the cultivation of regional brands, and strengthen the supervision of brand access, to win the satisfaction and trust of consumers, vigorously improve the popularity of regional brands at home and abroad and plan the working path for the enhancement of BC.
(2) The total indirect effect of ID on BC ranks second, which reflects the essence of improving regional BC. Although it has no direct impact on BC, the ID has a positive and important indirect impact on BC through the mediating role of BM and GG. This reminds us that only by strengthening the construction of various elements of the whole industrial chain, especially by establishing, perfecting, and popularizing the quality standard system of the industry, giving full play to the leading role of leading enterprises, implementing strict quality supervision and increasing the development and technological innovation of new products, can we enhance the market influence of the brand and provide a lasting driving force for the regional BC of characteristic agricultural products.
(3) GG is the key to enhancing the regional BC of characteristic agricultural products. The direct influence coefficient of GG on BC, ranking third among the influence factors. This reflects the respondents’ affirmation of the government’s efforts to strengthen support for characteristic agriculture, and they are full of expectations for the government to increase policy support further. GG also plays an important mediating role between RR, ID, and BC. It will play an important guiding role in improving the competitiveness of regional brands to strengthen the top-level design and industrial planning of the development of characteristic agricultural industry, increase the guiding role of policies, regulations, and fiscal and tax support, to encourage the industry associations to strengthen the integration of regional brands and the supervision of the quality of regional brand products, and further to improve the all-round service of the characteristic agricultural industry.
(4) RR is the foundation of the regional BC of characteristic agricultural products. RR has a positive mediating effect on BC through the mediating effect of BM and GG, and the mediating effect truly reflects the impact of RR on regional BC. This reminds us that only by unswervingly adhering to the protection of the natural and ecological environment of the production area of characteristic agricultural products, adhering to the protection of the green and environmental protection quality. Furthermore, by excavating the historical and cultural heritage of characteristic agricultural products, we can endow the regional BC of characteristic agricultural products with lasting vitality.

### Countermeasures and suggestions

Based on the above conclusions, this study puts forward the following countermeasures and suggestions for improving the regional BC of traditional advantageous characteristic agricultural products:

(1) The government should give full play to the leading role of the regional agricultural brand construction, mobilize the social resources of all walks of life, and strengthen the regional brand construction of characteristic agricultural products. The leaders need to understand that due to the “Matthew Effect” of brands and the “Entrenchment Effect” of strong brands of agricultural products around the globe. It is difficult for weak regional brands of agricultural products in developing countries to grow up entirely on their own in a purely market-oriented environment. Therefore, the help and support of the government become very important. The stories of Korea Guan Zhuang Commune and the DINZ of New Zealand just confirm the key role of GG.
(2) The construction of regional brands of agricultural products should be promoted with new international cooperation. In the final analysis, ID depends on the self-regenerating function of the agricultural industry in developing countries. While increasing material assistance to agriculture in developing countries, leadership from developed countries should keep in mind that since “it is better to teach others to fish than to give them fish,” they should help developing countries establish a characteristic agricultural industry system and set up a quality standard system of traditional advantageous characteristic agricultural products.
(3) The role of a “bridge” of trade associations should be strengthened. As a bridge between individuals and the government, as well as between all participants in brand construction, trade associations should play the role of market cooperation. It represents the interests of enterprises and leaders, coordinates the interests of all parties involved, supervises product quality, provides market information, offers guidance of production technology, assists in policy implementation, expands the international market and so on.
(4) The leading role of key leaders and the support of the industrial chain should be reinforced. The developing countries and underdeveloped agricultural countries are at the low end of the agricultural value chain. It is necessary to build an agricultural, industrial chain based on the regional brand of special agricultural products, drawing upon the advantageous resources, integrate small farmers into the industrial chain, and establish an agribusiness, which will play a leading role in developing the regional brand of agricultural products and form a competitive industrial cluster.
(5) The regional brands of traditional advantageous characteristic agricultural products should be empowered with traditional culture. An important part of RR capacity consists of traditional cultural resources, whereas culture is one of the basic elements of the brand. Every nation has its splendid civilization, where the traditional advantageous characteristic agricultural products are often the carrier and even an emblem of its traditional culture. Then exploiting the value of regional historical and cultural resources is an effective way to build regional brands of agricultural products.
(6) Efforts should be made to increase farmers’ income by constructing regional brands of agricultural products. Economic development should be agricultural products into an important way to make farmers rich, organize farmers in better ways and introduce agricultural cooperative organizations into the region so that farmers can fully participate in the activities that will bring them real benefit and stimulate their enthusiasm to join in the cause of achieving common prosperity.

### Limitations and future research

In terms of research limitations, the sample of this study is not large enough, for only two typical industries of ginseng and sika deer in Jilin, China was investigated. Therefore, it is suggested that more industries (say more than 3 typical industries) should be selected for future research. Also, larger samples may be taken in multiple countries, to increase the generalization of the research results. Furthermore, the literature is based on Chinese context and research; therefore, future scholars are advised to study from different national perspectives or to make global comparisons on specific topics.

## Data availability statement

The raw data supporting the conclusions of this article will be made available by the authors, without undue reservation, to any qualified researcher.

## Ethics statement

Ethical review and approval was not required for the study on human participants in accordance with the local legislation and institutional requirements. Written informed consent for participation was not required for this study in accordance with the national legislation and the institutional requirements.

## Author contributions

PL: conceptualization, methodology, data curation, and writing—original draft preparation. JD: supervision, fund acquisition, and project administration. FS: software, formal analysis, and writing—review and editing. All authors equally contributed to revising and finalizing the manuscript.

## Conflict of interest

The authors declare that the research was conducted in the absence of any commercial or financial relationships that could be construed as a potential conflict of interest.

## Publisher’s note

All claims expressed in this article are solely those of the authors and do not necessarily represent those of their affiliated organizations, or those of the publisher, the editors and the reviewers. Any product that may be evaluated in this article, or claim that may be made by its manufacturer, is not guaranteed or endorsed by the publisher.
